# House Hunters, Gerontology Style: A Unique Classroom Activity for Undergraduates

**DOI:** 10.1093/geroni/igab046.742

**Published:** 2021-12-17

**Authors:** Meghan McDarby

**Affiliations:** Washington University, St. Louis, Missouri, United States

## Abstract

Small group discussion activities that capitalize on students’ interest in technology may generate enthusiasm for course content in gerontology. We describe a unique classroom activity that supports discussion about retirement issues in older adulthood by leveraging student dexterity in utilizing web applications. In this activity, students act as real estate agents for a retired older adult couple who is relocating to be closer to family. Students are presented with details about the couple, including demographic information (e.g., age, functional limitations, hobbies) and the couple’s “wish list” for features and amenities of their future home. Then, students use these details to choose a home for the couple on Zillow and prepare a “pitch” of the home that is presented to the class and judged by the course instructor. Feedback from students suggests that this activity offers a “real world application to course material” and facilitates enthusiasm about course content.

